# Disrupted NAD(P) Metabolism and Xanthine Dehydrogenase in a Stress-Induced Rat Model of Depression: NMR Metabolomics Insights

**DOI:** 10.3390/metabo14120660

**Published:** 2024-11-27

**Authors:** Songjiao Chen, Jumeng Wei, Yongchi Wang, Yidan Yao, Haibo Wang, Jie Peng, Jinquan Li

**Affiliations:** 1 College of Resources and Environment Science, Anhui Science and Technology University, Fengyang 233100, China; 2 School of Life and Health Science, Anhui Science and Technology University, Fengyang 233100, China; 3 Innovative Institute of Animal Healthy Breeding, College of Animal Science and Technology, Zhongkai University of Agriculture and Engineering, Guangzhou 510225, China

**Keywords:** uric acid, NAD(P) deficiency, xanthine oxidoreductase, untargeted metabolomics, rat model of depression

## Abstract

**Background**: Clinical findings have shown a negative correlation between the severity of depressive symptoms and serum uric acid levels in men, yet the role of metabolic regulation in the pathophysiology of depression remains largely unknown. **Methods**: In this study, we utilized an acute restraint-stress-induced male rat model of depression to investigate biochemical changes through NMR-based metabolomics combined with serum biochemical analysis. Additionally, we employed qPCR, immunoblotting, and enzyme activity assays to assess the expression and activity of xanthine oxidoreductase, the rate-limiting enzyme in uric acid production. **Results**: Our findings indicate the following: (1) restraint stress is a valid method for inducing a depressive phenotype in rats; (2) depressive rats exhibit decreased NAD(P) levels in the liver and increased nicotinamide *N*-oxide and nicotinate levels in urine, accompanied by decreased levels of uric acid, allantoin, and allantoic acid in serum or tissues; (3) xanthine dehydrogenase activity is diminished in depressive rats without corresponding changes in gene or protein expression. **Conclusion**: The increased urinary excretion of NAD(P) precursors results in reduced hepatic NAD(P) levels, thereby suppressing NAD-dependent xanthine dehydrogenase activity and diminishing the production of uric acid and its downstream metabolites (allantoin and allantoic acid).

## 1. Introduction

Depression, a common mood disorder, is one of the leading causes of disability worldwide and places a heavy burden on society and families [1]. Enormous strides have been made in developing drugs to prevent and treat depression; however, the therapeutic outcome remains inconsistent. For example, about 35% of patients treated with selective serotonin reuptake inhibitor (SSRI) achieve remission, but the vast majority of remitted individuals (~85%) will experience symptom recurrence [2,3]. Previous reports have shown that metabolic correction can improve depressive symptoms or lead to remission [4]. Furthermore, clinical evidence indicated that symptom severity and duration of depression are negatively correlated with serum uric acid levels in men but not in women [5,6,7,8]. Nevertheless, the underlying gender-specific mechanisms and the relationships between metabolism, redox, and depression have not yet been fully explored. Therefore, it is crucial to investigate the metabolic underpinnings of depression systematically to identify key sex-specific disease-causing factors and develop more effective treatments.

In the past few decades, various stress-based animal models have emerged to support the pathophysiological studies of depression and drug-screening studies of potential antidepressants. The restraint stress protocol is one of the most commonly used procedures to induce stress-related depression [9], capable of inducing both acute and chronic stress. In the acute restraint stress protocol, animals are typically confined in a cylindrical tube with ventilation holes for 120–180 min. Twenty-four hours post-stress, animals show high depression-like behavior, such as reduced locomotor activity and food intake. Restraint-stress-induced hypolocomotion may reflect an inability or unwillingness to explore unfamiliar surroundings, indicating a failure to adapt to stress. Earlier studies have shown that acute stress leads to a rapid increase in mRNA levels of stress-related receptors at 3–8 h post-stress, returning to baseline levels at 48 h post-stress. However, at 48 h post-stress, plasma adrenocorticotropic hormone (ACTH) and corticosterone levels are not fully recovered [10,11]. Therefore, examining the biochemical signatures within biofluids and tissues of male animals at 48 h post-exposure to stress could provide valuable insights into the mechanisms underlying stress-induced, gender-specific depression.

The present study focused on clarifying the biochemical changes in the acute restraint-stress-induced male rat model of depression using ^1^H NMR-based metabolomics combined with serum biochemical analysis, while exploring the underlying metabolic alterations and their impact on uric acid metabolism in the pathophysiology of depression.

## 2. Materials and Methods

### 2.1. Animal Handling

All animal procedures in this study were conducted in compliance with the National Institutes of Health Guide for the Care and Use of Laboratory Animals and were reviewed and approved by the Anhui Laboratory Animal Care Committee. The study utilized seven-week-old male Sprague-Dawley (SD) rats, weighing 200 ± 10 g, obtained from Beijing Vital River Laboratory Animal Technology Co., Ltd., Beijing, China. These rats were kept in a controlled environment with the following parameters: temperature maintained at 24 ± 2 °C, relative humidity between 40% and 60%, and a 12 h light/dark cycle. They had unrestricted access to food and water, and their body weight was recorded daily. After a one-week acclimatization period, 13 male rats (mean weight: 241 ± 15 g) were randomly assigned to two groups: a non-stressed control group (*n* = 6) and a stressed group (*n* = 7). In the acute restraint stress protocol, rats in the stressed group were individually placed in ventilated plastic restrainers without food or water for 120 min, as described in previous studies [12,13], while control rats remained undisturbed in their home cages with social contact but no access to food or water, following standard procedure.

### 2.2. Open Field Test

Both non-stressed and stressed rats were given a 30 min undisturbed period prior to undergoing the open field test. During the test, each rat was individually placed in a corner of the peripheral area of a square box (90 cm in length, 90 cm in width, and 40 cm in height) and allowed to explore freely for 10 min. The box was divided into a 20 cm wide peripheral zone, known as the gallery, and a central zone occupying approximately 40% of the total area. It was illuminated by a 40 W reflector lamp. The rats’ behavioral traits were assessed using metrics such as the distance traveled and the duration spent in both the peripheral and central zones [14]. Following each test, the box was sanitized with 70% ethanol to ensure cleanliness for subsequent rats.

### 2.3. Sample Collection

Urine sample collection procedure: Rats were individually placed in metabolic cages for urine collection over designated two-hour periods. The baseline (0 h) sample was collected from −2 to 0 h, while post-stress samples were taken at 22 to 24 h (24 h sample) and 46 to 48 h (48 h sample). In total, 20 urine samples were obtained from 13 rats: 6 control rats provided urine at the 0 h time point, and 7 stress-treated rats provided samples at both the 24 h and 48 h marks. Each sample was collected into a pre-chilled 5 mL tube containing 0.1 mL of a 1% NaN_3_ solution to inhibit bacterial growth. Following collection, samples were snap-frozen using liquid nitrogen and stored at −80 °C for preservation.

Blood sample collection: Rats were euthanized through exsanguination under isoflurane anesthesia 48 h post-stress. Blood samples were then divided into two portions: one portion was kept at 4 °C overnight to allow serum preparation for subsequent biochemical analysis, while the other portion was processed in a heparinized tube and centrifuged to obtain plasma for NMR spectroscopy.

Tissue sample collection: Following euthanasia, tissues from the liver, kidney, spleen, lung, and brain were harvested in duplicate. One set of tissues was fixed in 10% formalin (*v*/*v*) for histopathological analysis, while the other set was rapidly frozen using liquid nitrogen and stored at −80 °C for subsequent tissue extraction and NMR analysis.

### 2.4. Histopathology

Tissue samples from the kidney, liver, lung, and spleen of both non-stressed control and stressed rats were fixed in 10% formalin. Following fixation, the tissues were embedded in wax, sectioned at 5 μm thickness, and stained with hematoxylin and eosin for microscopic analysis.

### 2.5. ACTH and Corticosterone Measurements and Serum Biochemical Analysis

ACTH and plasma-free corticosterone levels were quantified using an ACTH ELISA kit (MD Bioproducts) and a corticosterone ELISA kit for rats (Labor Diagnostika Nord), respectively, following the manufacturer’s instructions. Absorbance was measured at 450 nm (with a reference wavelength of 630 nm) using a microplate reader (Tecan, Mannedorf, Switzerland).

Serum chemical analysis was conducted using a Roche Modular P800 automatic analyzer (Roche Diagnostics, Mannheim, Germany) to measure a variety of biomarkers, including total protein (TP), albumin (Alb), globulin (Glo), Alb/Glo ratio, total bilirubin (Tbil), indirect bilirubin (Ibil), direct bilirubin (Dbil), aspartate aminotransferase (AST), alanine aminotransferase (ALT), AST/ALT ratio, alkaline phosphatase (ALP), gamma-glutamyltransferase (GGT), glucose (Glu), total cholesterol (TC), triglycerides (TG), high-density lipoprotein (HDL), low-density lipoprotein (LDL), lactate dehydrogenase (LDH), uric acid (UA), creatinine (Cn), blood urea nitrogen (BUN), BUN/Cn ratio, and total bile acid (TBA). All results are presented as the mean ± standard error of the mean (SEM).

### 2.6. Sample Preparation and ^1^H NMR Spectroscopic Analysis

For plasma samples, an aliquot of 300 μL was mixed with 300 μL of phosphate D_2_O buffer solution (K_2_HPO_4_/NaH_2_PO_4_, 60 mM, pH 7.4). After mixing and centrifuging at 10,000× *g* at 4 °C for 10 min, an aliquot (500 μL) of supernatant was transferred to 5 mm NMR tubes and the potential metabolic profile was analyzed by NMR. The NMR experiment was completed on a Varian INOVA 500 MHz at 296 K. ^1^H NMR spectra of plasma samples were acquired with a CPMG pulse sequence (RD-90°-{τ-180°-τ}_n_-ACQ), with a recycle delay (RD) of 1 s, an echo delay τ of 230 μs, and the n of 100. The free induction decays (FIDs) were collected into 40 K data points over a spectral width of 10 kHz, and 64 scans were accumulated for each spectrum.

For urine samples, an aliquot of 455 μL was mixed with 55 μL of D_2_O buffer solution (K_2_HPO_4_/NaH_2_PO_4_, 1.5 M, containing 0.1% sodium 3-(trimethylsilyl) propionate-2,2,3,3-d_4_ (TSP), pH 7.4) to minimize the pH fluctuation of urine samples. The mixture was vortexed and centrifuged at 10,000× *g* for 10 min at 4 °C. An aliquot (500 μL) of supernatant was transferred to a 5 mm NMR tube and the metabolic characteristics were analyzed with NMR spectroscopy. ^1^H NMR spectra were acquired on a Varian INOVA 500 MHz spectrometer at 296 K. ^1^H NMR spectra of urine samples were acquired with a NOESYPR1D pulse sequence (RD-90°-τ_1_-90°-τ_m_-90°-ACQ), with a recycle delay (RD) of 1 s, τ_1_ of 5 μs, and τ_m_ of 80 ms. FIDs were collected into 52 K data points over a spectral width of 10 kHz, and 64 scans were accumulated for each spectrum.

Polar metabolites from rat tissue samples were extracted using a method previously described in our study [15]. Briefly, 100 mg of tissue (brain, liver, kidney, spleen, or lung) was homogenized in 400 μL of methanol (CH_3_OH) and 85 μL of water (H_2_O) at 4 °C. To the homogenate, 400 μL of chloroform and 200 μL of water were added, followed by vortex mixing. The mixture was then centrifuged at 10,000× *g* for 5 min at 4 °C. The upper aqueous layer was carefully separated and dried to remove water and solvents. The resulting dried extract was reconstituted in 0.5 mL of deuterium oxide (D_2_O) containing 1 mM of sodium 3-trimethylsilylpropionate (TSP) for NMR analysis. Proton NMR spectra were recorded on a Bruker AVII 600 MHz spectrometer at 296 K, using a solvent-suppressed ZGPR pulse sequence (RD-90°-ACQ) with a 6.5 μs relaxation delay. Data were collected in 64 K points over a spectral width of 12 kHz, and 64 scans were averaged for each spectrum.

### 2.7. Spectral Processing and Pattern Recognition

The NMR spectra obtained were processed for phase and baseline correction using MestReNova (V9.0, Mestrelab Research, Galicia, Spain). Reference deconvolution was performed using the CH3 signal of lactic acid at δ 1.33 for plasma samples, and the TSP signal at δ 0.00 for tissue and urine extracts.

^1^H NMR spectra of plasma, urine, or tissue extracts were segmented into bins with a width of 0.005 ppm using the AMIX package (v.3.8). The segments δ 5.90–5.40 and δ 5.10–4.20 in the plasma spectra and the segments δ 5.90–5.35 and δ 5.25–4.14 in the urine spectra were removed to exclude the urea signal (δ 5.5–6.1 ppm) and the residual water signal (δ 4.50–4.9 ppm). For the spectra of tissue extracts, the segments δ 5.22–4.67 and δ 3.40–3.31 were removed to exclude the residual water and methanol signals.

To ensure comparability across samples, data normalization was applied to rescale the integral values of the spectra, facilitating subsequent statistical analysis. For quality verification of the spectra, unsupervised principal component analysis (PCA) with mean-centered scaling was employed. To identify group differences, supervised orthogonal partial least squares discriminant analysis (OPLS-DA) with Pareto scaling was conducted. The score plots were used for optimal 2D visualization of the samples, while the corresponding coefficient loading plots from OPLS-DA highlighted the impact of variables on the scores. A Pearson correlation coefficient |r| > 0.755 (with degrees of freedom = 5) was adopted as the threshold for significance, ensuring statistically meaningful outcomes at *p* < 0.05.

### 2.8. Gene Expression Level of Xanthine Oxidoreductase (XOR) in Liver Tissues

According to the manufacturer’s instructions, the total RNA of liver samples was isolated using a Trizol RNA extraction kit (Takara, Cat.9109; Osaka, Japan). Total RNA was reverse-transcribed into cDNAs using a PrimeScript RT reagent kit with gDNA Eraser (RR047A, Takara). Quantitative real-time PCR was performed using AceQ qPCR SYBR Green Master Mix (Q131, Vazyme, Nanjing, China) with an initial denaturation at 95 °C for 30 s, followed by 40 cycles of 95 °C for 30 s and 72 °C for 30 s. The sequences of primers used for RT-qPCR are as follows: XOR forward primer, 5′-AGAGGGCCATCTATGCATCC-3′; XOR reverse primer, 5′-CACAGGCGTTTCGGATCTTC-3′; GAPDH forward primer, 5′-GGCACAGTCAAGGCTGAGAATG-3′; GAPDH reverse primer, 5′-ATGGTGGTGAAGACGCCAGTA-3′. The relative mRNA abundance was calculated using the 2^−ΔΔCt^ method compared to the housekeeping gene GAPDH.

### 2.9. The Protein Expression Level of Xanthine Dehydrogenase (XDH) in Liver Tissues

Liver lysates were prepared using RIPA buffer, with protein concentrations determined via the Bradford assay. Equal amounts of protein were loaded onto a 6% SDS-PAGE gel for electrophoresis and subsequently transferred to a PVDF membrane. Detection of xanthine dehydrogenase (XDH, 150 kDa), xanthine oxidase (XO, 130 kDa), and a primary cleavage fragment (85 kDa) was conducted using a rabbit monoclonal antibody (Abnova, Catalog No. MAB22989; Hsinchu City, Taiwan, China), while β-actin was detected using a monoclonal antibody (Santa Cruz Biotechnology, Catalog No. sc-130656; Santa Cruz, CA, USA). The membrane was incubated with HRP-linked secondary antibodies for 1 h at room temperature. Protein bands were visualized with the Clarity Western ECL substrate (Bio-Rad Laboratories, Catalog No. 170-5060; Hercules, CA, USA).

### 2.10. Enzyme Activity of XOR

XOR, an essential enzyme in uric acid synthesis, exists as xanthine dehydrogenase (XDH) and xanthine oxidase (XO), each using distinct electron acceptors. While XDH primarily utilizes NAD, XO preferentially engages oxygen (O_2_) as the electron acceptor. The activities of XOR and XO in serum were quantified by tracking absorbance at 290 nm, with xanthine as the substrate. XOR activity was assessed in the presence of NAD, and XO activity was determined without NAD. XDH activity was then derived by subtracting XO activity from total XOR activity.

The protocol involved preparing a working solution containing 50 mM potassium phosphate buffer (pH 7.8), 0.1 mM xanthine, 10 mM EDTA, 1 mM dithiothreitol, 0.5 mg/L leupeptin, and 1 mM phenylmethylsulfonyl fluoride. For baseline readings, 10 μL of water was combined with 250 μL of the working solution, and 200 μL was transferred to a 96-well plate. Absorbance at 290 nm was recorded at the start and after 30 min. For the test readings, 10 μL of serum sample was mixed with 250 μL of the working solution with (XOR) or without (XO) 0.5 mM NAD^+^, with absorbance measured similarly. The absorbance change (Δ*A*) was calculated for both baseline and test samples. Enzyme activity, reported in units per milliliter (U/mL), was defined as the amount of enzyme needed to catalyze the formation of 1 μM of uric acid per minute. The final activity was determined using the following formula:

$$Enzyme\;activity\left(\frac{U}{mL}\right)=\frac{\left(∆A_{test}-A_{blank}\right)\times V_{total}}{\epsilon \times d\times V_{sample}\times T}\times 10^{6}$$

where *V_total_* = 2.6 × 10^−4^ L (total reaction volume), *ε* = 1.22 × 10^4^ L/mol/cm (molar extinction coefficient of uric acid), *d* = 0.6 cm (optical path length of the 96-well plate), *V_sample_* = 0.01 mL (volume of sample added), *T* = 30 min (reaction time). To ensure assay consistency, the same substrate concentrations, reaction conditions, and time intervals were used across all samples. Additionally, all enzyme activity measurements were conducted in triplicate for each sample, and the average value was used in the final analysis.

### 2.11. Statistical Analysis

Statistical analyses were conducted using the Statistical Package for Social Sciences (SPSS) version 22.0. To assess significant differences between the control and experimental groups, an unpaired two-tailed Student’s *t*-test was applied. Statistical significance was defined as a *p*-value below 0.05. Data are expressed as mean ± SEM.

## 3. Results

### 3.1. Acute Restraint Stress Significantly Reduces Only the Tissue-To-Body Weight Ratio of the Brain and Has Limited Effects on the Gross Histological Morphology of All Tested Tissues

**Figure 1:**
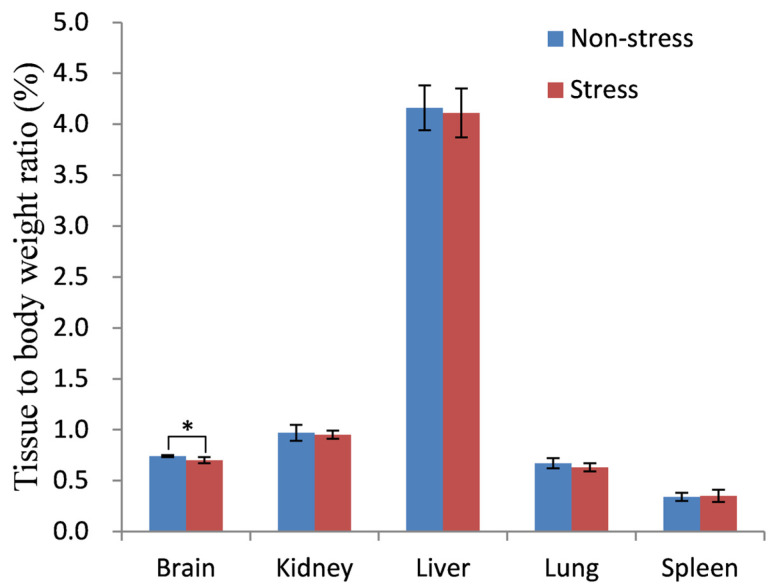
Tissue-to-body weight ratios (%). Non-stress, non-stressed control group; Stress, stressed group. * represents a significant difference between the two groups as determined by Student’s *t*-test (* *p* < 0.05).

Weight fluctuations are often associated with depression. The tissue-to-body weight ratio serves as a reliable metric to detect stress-induced weight changes, potentially preceding visible morphological alterations [16]. In this study, we evaluated tissue weight and morphology following acute restraint stress using tissue-to-body weight ratios and histological staining. As illustrated in Figure 1, no statistically significant differences were observed in the tissue-to-body weight ratios for all examined tissues, except for the brain. The brain-to-body weight ratio in stressed rats was notably lower than in non-stressed controls, with a significant *p*-value of 0.018 (*p* < 0.05). Additionally, no significant body weight differences were found between the stressed and control groups (see Supplementary Table S1). Histological analysis also revealed no discernible changes in the kidney, lung, liver, and spleen tissues of stressed versus non-stressed rats (Supplementary Figure S1). These findings indicate that the brain exhibits greater sensitivity to stress compared to other tissues.

### 3.2. Acute Restraint Stress Significantly Reduces Moving Distance in Open Field Test but Has Limited Effects on ACTH and Corticosterone Levels at 48 h Post-Stress

**Figure 2:**
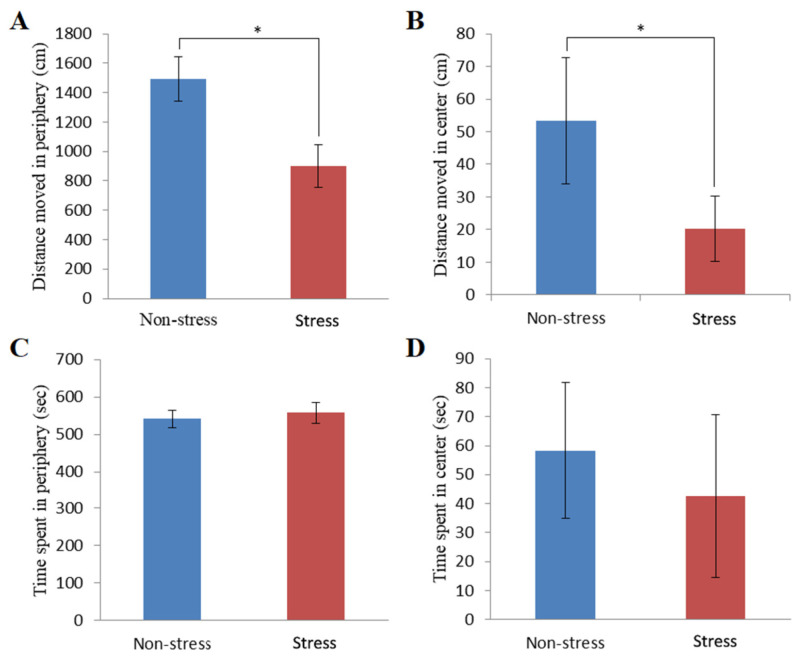
Behavioral response of non-stressed and stressed rats in the open field apparatus. (**A**) Total distance moved in the peripheral zone of the open field. (**B**) Distance moved in the center zone of the open field. (**C**) Time spent in the peripheral zone of the open field. (**D**) Time spent in the center zone of the open field. Non-stress, non-stressed control group; Stress, stressed group. The values are expressed as means ± S.E.M. * indicates a significant difference between the two groups as determined by Student’s *t*-test (* *p* < 0.05).

The open field test evaluated rats’ depression-like behavior (a test for locomotor activity and novelty exploration). The activities of stressed rats exhibited a significant decrease in the moving distances (899 ± 144 cm in the periphery and 20 ± 10 cm in the center), as compared to those in the control group (1494 ± 153 cm in the periphery and 58 ± 24 cm in the center) (Figure 2A,B). However, the duration times in the peripheral (43 ± 28 s versus 58 ± 24 s) and central (557 ± 28 s versus 542 ± 24 s) areas showed no significant differences between the non-stressed and stressed rats (Figure 2C,D). These data show that stress exposure resulted in a significant decrease in locomotor activity, which is concordant with the characteristics of depression.

**Figure 3:**
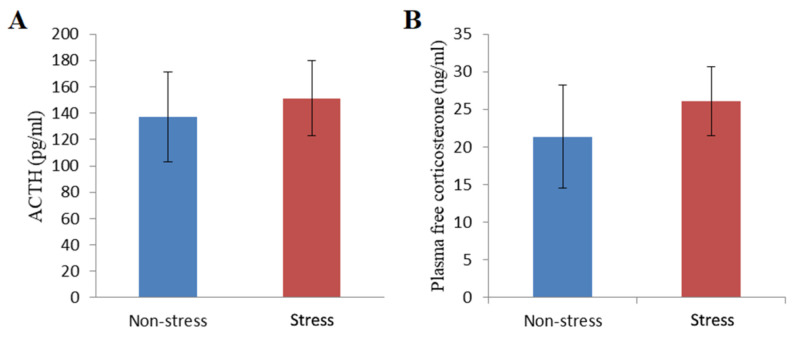
ACTH values and plasma-free corticosterone values in non-stressed control and stressed rats. (**A**) Measurement of ACTH level of non-stressed and stressed rats using the ELISA test. (**B**) Measurement of plasma-free corticosterone level of non-stressed and stressed rats using the ELISA test. Non-stress, non-stressed control group; Stress, stressed group. The values are expressed as means ± S.E.M.

In addition to behavioral measures, to understand the role of the HPA axis in stress-induced depression-like behavior, two stress hormones, ACTH and corticosterone, were determined in non-stressed and stressed rats at 48 h post-stress. As shown in Figure 3A,B, there were no statistically significant differences in ACTH level (137.32 ± 34.07 pg/mL for the non-stressed control group versus 151.26 ± 28.69 pg/mL for the stressed group) and corticosterone level (21.34 ± 6.86 ng/mL for the non-stressed control group versus 26.09 ± 4.62 ng/mL for the stressed group). These data suggest that the recovery of ACTH and corticosterone levels is nearing completion.

### 3.3. Acute Restraint Stress Increases Ibil Levels and Decreases Glucose and Uric Acid Levels in Serum Biochemical Tests

The serum contains various biomolecules, including proteins, enzymes, lipids, metabolites, and nucleic acids. The measurement of these biomolecules provides information about the body’s functional status. In this study, serum biochemical parameters including TP, Alb, Glo, Alb/Glo, Tbil, Dbil, Ibil, ALT, AST, AST/ALT, GGT, ALP, TG, TC, HDL, LDL, Glc, LDH, BUN, Cn, Bun/Cn, UA, and TBA were used to evaluate the effects of acute restraint stress on rats. As shown in Table 1, the Ibil level in the serum of stressed rats (1.28 ± 0.15 μmol/L) was significantly higher than that of the non-stressed control group (1.00 ± 0.20 μmol/L), with *p* < 0.05. As a breakdown product of heme, the rise in Ibil suggests dysregulation of heme metabolism. The decreased glucose level may mean that the energy metabolism is affected by stress.

In addition, another noteworthy change was the downregulation of the uric acid level in the serum (164.67 ± 78.32 μmol/L for the non-stressed control group versus 104.58 ± 26.78 μmol/L for the stressed group), though this difference was not statistically significant (*p*-value = 0.082). The lack of significance could be attributed to within-group variation.

### 3.4. ^1^H NMR Spectral Profiles and Characteristics of Body Fluids and Tissues from Rats

**Figure 4:**
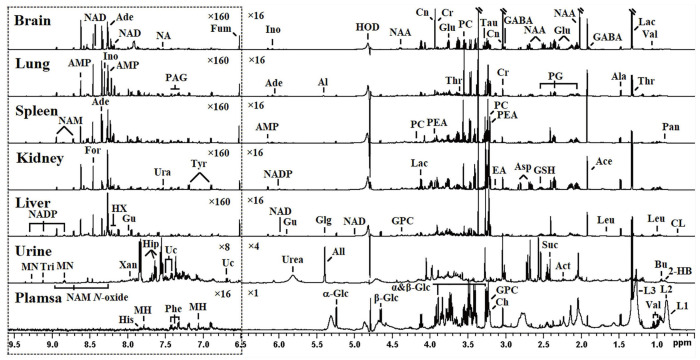
The representative ^1^H NMR spectra of plasma, urine, and aqueous extracts from the liver, kidney, spleen, lung, and brain tissues of both non-stressed and stressed rats. The spectrum’s region δ6.5–9.6 (in the dotted box) was magnified 16, 2, or 10 times in vertical expansion compared with the region δ0.5–6.5. Keys: Ace, Acetate; Act, Acetone; Ade, Adenosine; Ala, Alanine; Al, Allantoate; All, Allantoin; AMP, Adenosine monophosphate; Asp, Aspartate; Bu, Butyrate; Ch, Choline; CL, Cholate; Cn, Creatinine; Cr, Creatine; EA, Ethanolamine; For, Formate; Fum, Fumarate; GABA, *γ*-Aminobutyrate; Glg, Glycogen; *α*-Glc, Alpha-glucose; *β*-Glc, Beta-glucose; Glu, Glutamate; GPC, Glycerophosphocholine; GSH, Glutathione; Gu, Guanosine; 2-HB, 2-Hydroxybutyrate; Hip, Hippurate; His, Histidine; HX, Hypoxanthine; Ino, Inosine; L1, LDL, CH_3_-(CH_2_)_n_-; L2, VLDL, CH_3_-(CH_2_)_n_-; L3, LDL, CH_3_-(CH_2_)_n_-; Lac, Lactate; Leu, Leucine; MH, 1-Methylhistidine; MN, 1-Methylnicotinamide; NA, Nicotinate; NAM, Nicotinamide; NAM *N*-oxide, Nicotinamide *N*-oxide; NAA, *N*-Acetylaspartate; NAD, NAD^+^; NADP, NADP^+^; Pan, Pantothenate; PC, Phosphocholine; PEA, Phosphoethanolamine; Phe, Phenylalanine; Suc, Succinate; Tau, Taurine; Thr, Threonine; Tri, Trigonelline; Tyr, Tyrosine; Uc, Urocanate; Ura, Uracil; Val, Valine; Xan, Xanthine.

In the ^1^H NMR spectra of body fluids (urine and plasma) and tissue extracts (brain, lung, spleen, kidney, and liver) from stressed rats (Figure 4), primary peaks were identified and matched to specific metabolites as detailed in Supplementary Table S2, drawing on previous research [15,17]. The metabolites detected in the NMR spectra spanned a range of compound classes, including organic acids, carbohydrates, amino acids, nucleotides, and various intermediates and end products of metabolic pathways. Each type of tissue exhibited a distinctive spectral profile, offering insights into the biochemical changes occurring within.

**Figure 5:**
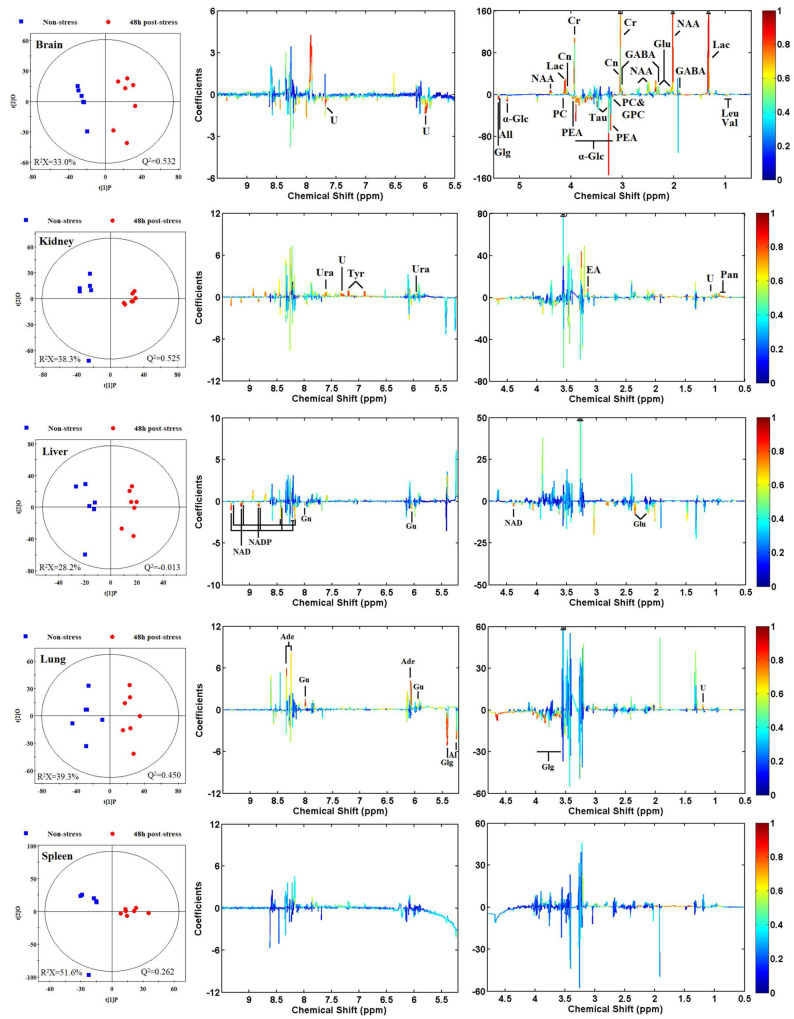
OPLS-DA score plots (**left**) and OPLS-DA loadings plots (**middle** and **right**) derived from the ^1^H NMR data of brain, kidney, liver, lung, and spleen tissues. Non-stress, non-stressed control group; 48 h post-stress, stressed group. Q^2^ and R^2^X indicate the predictability and interpretability of the model, respectively. A warm color represents a significant difference between classes, and a cool color represents no significant difference. The abbreviations are consistent with the annotations in Figure 4, and the detailed assignment information is listed in Table 2 and Supplementary Table S2.

**Figure 6:**
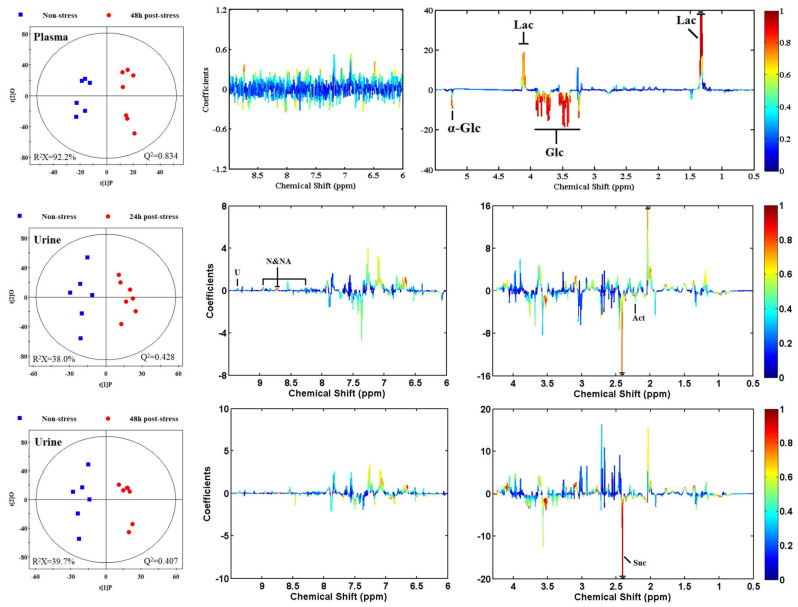
OPLS-DA score plots (**left**) and OPLS-DA loadings plots (**middle** and **right**) were derived from the ^1^H NMR data of plasma and urine samples.

Unsupervised principal component analyses (PCA) were conducted on the respective NMR datasets to uncover comprehensive metabolic insights and scrutinize the inherent variability within the tissues in question. Intriguingly, the PCA outcomes revealed a striking distinction between brain extracts and plasma samples when compared to their corresponding control samples (see Supplementary Figure S2 for details). To further delineate the metabolic disparities between non-stressed and stressed rats, supervised orthogonal-partial-least-squares-discriminant-analysis (OPLS-DA) models were formulated based on the NMR datasets. The OPLS-DA score plots (depicted in the left panels of Figure 5 and Figure 6) vividly illustrate a clear segregation between the treated groups and their respective controls. Meanwhile, the OPLS-DA loading plots (displayed in the middle and right panels of Figure 5 and Figure 6) provide a granular view of the metabolites driving this segregation, as determined by the first principal component. For instance, in the plasma loading plot (Figure 6), the prominent negative signals of glucose, colored in red, denoted a substantial decline in glucose levels among the stressed group. Conversely, in the brain loading plot (Figure 5), metabolites with positive signals depicted in warm hues signified elevated levels in the brains of stressed rats, and vice versa. These fluctuations in metabolites mirrored the biochemical adjustments in response to acute restraint stress. A comprehensive tabulation of the significant class-discriminating metabolites across all pairwise groups is presented in Table 2.

Non-stress, non-stressed control group; 48 h post-stress, stressed group. Q^2^ and R^2^X indicate the predictability and interpretability of the model, respectively. A warm color represents a significant difference between classes, and a cool color represents no significant difference. The abbreviations are consistent with the annotations in Figure 4, and the detailed assignment information is listed in Table 2 and Supplementary Table S2.

### 3.5. Metabolomic Responses of Tissues and Body Fluids from Rats to Acute Restraint Stress Treatments

According to the correlation coefficient, 30 discriminating metabolites were identified as the key metabolites responsible for class separation. In the brain metabolome, levels of creatine, creatinine, *γ*-aminobutyrate (GABA), glutamate, lactate, and *N*-acetyl aspartate (NAA), were significantly upregulated, while levels of allantoin, *α*-glucose, glycerophosphocholine (GPC), glycogen, leucine, NAD, phosphocholine, phosphoethanolamine (PEA), taurine, threonine, and valine were significantly downregulated (Table 2). Similarly, levels of four metabolites, including ethanolamine, pantothenate, tyrosine, and uracil, were increased in the kidney metabolome, while levels of five metabolites, including allantoin, glutamate, guanosine, NAD, and NADP, were found to be decreased in the liver metabolome. In the lung metabolome, increased adenosine and guanosine levels and decreased allantoic acid levels were observed. In the plasma metabolome, an increase in lactate level and a decrease in glucose level were observed. In the urine metabolome, the nicotinamide *N*-oxide and nicotinic acid levels increased, while acetone and succinate levels decreased at 24 h and 48 h post-stress, respectively. However, there was no significant discriminating metabolite between non-stressed and stressed groups in the spleen metabolome (Table 2). These data once again suggest that the brain is more susceptible to stress than the other tissues.

### 3.6. Acute Restraint Stress Has Limited Effects on Gene Expression and Protein Abundance but Significantly Decreases the Enzyme Activity of XOR

**Figure 7:**
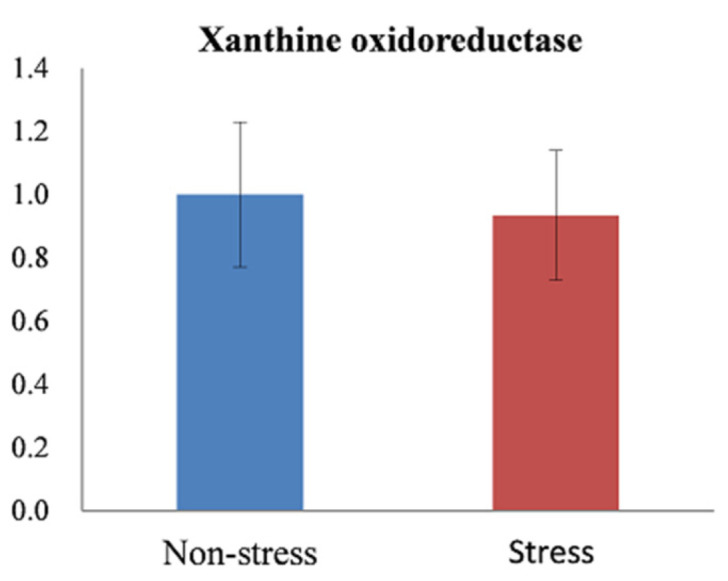
Gene expression levels of xanthine oxidoreductase in the livers of non-stressed control and stressed rats. Non-stress, non-stressed control group; Stress, stressed group.

**Figure 8:**
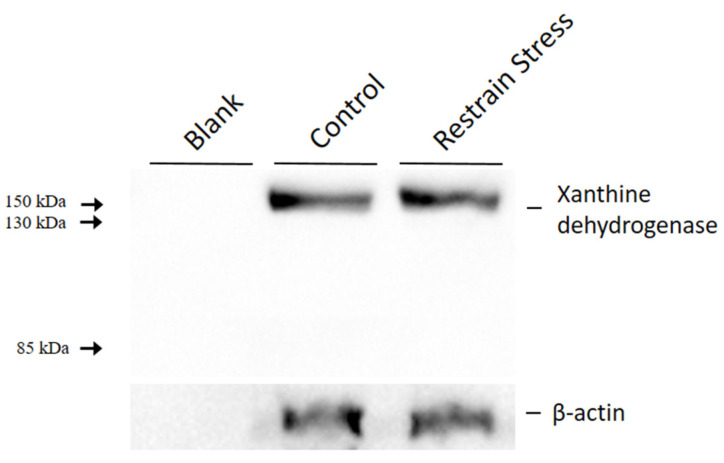
Protein expression levels of xanthine oxidoreductase in the livers of non-stressed control and stressed rats. Non-stress, non-stressed control group; Stress, stressed group.

In line with the decreased uric acid level in depression patients, acute restraint-stress-induced depression-like rats exhibited a non-statistical decrease in serum uric acid level and significant decreases in allantoin, a downstream metabolite of uric acid, in the liver and brain. According to these findings, the downregulation of uric acid biosynthesis in the liver may occur in acute restraint-stress-induced depression-like rats. To establish whether XOR, the rate-limiting enzyme for uric acid production, plays a role in uric acid metabolism in our rat model of depression, we employed qPCR and Western blot to determine the gene and protein expression of XOR in the liver. The results of qPCR (Figure 7) and Western blot analysis (Figure 8) showed that both gene and protein expression of XOR showed no significant differences between non-stressed and stressed rats. Furthermore, the 150-kDa XDH protein can be irreversibly proteolyzed into the 130-kDa form and further proteolyzed into an 85-kDa fragment with XO activity [18]. Considering that no band around 130 kDa and 85 kDa was detected by Western blot analysis (Figure 8), this may mean that acute restraint stress did not trigger the irreversible conversion of the substrate-reduced enzyme XDH to the oxidative enzyme XO.

XOR and XO activities were measured in serum from non-stressed and stressed rats. There was no statistically significant difference in xanthine oxidase activity (Supplementary Figure S3, 0.556 ± 0.091 U/mL for the non-stressed control group versus 0.523 ± 0.081 U/mL for the stressed group), but the XOR activity decreased significantly (Supplementary Figure S4, 0.802 ± 0.099 U/mL for the non-stressed control group versus 0.694 ± 0.079 U/mL for the stressed group, *p* < 0.05). The serum XDH activity, obtained by subtracting the mean XO activity from the mean XOR activity, was 0.246 U/mL for the non-stressed control group and 0.171 U/mL for the stressed group (Table 3). There was a decrease in XDH activity in the stressed group.

## 4. Discussion

**Figure 9:**
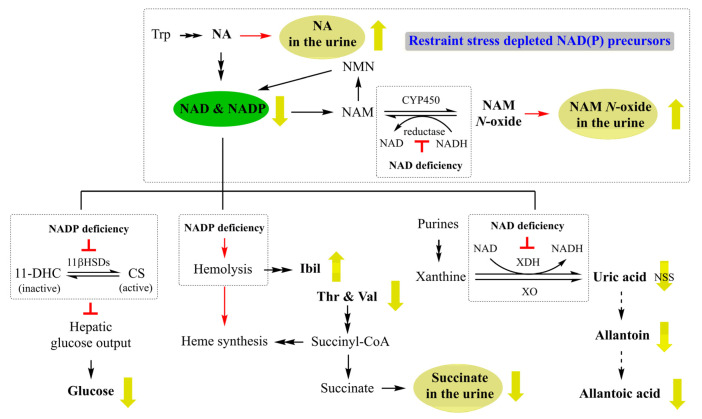
Schematic representation of the proposed mechanism for the roles of NAD depletion on metabolism dysfunction in an acute restraint-stress-induced rat model of depression. Green arrows indicate up- or down-regulation; red arrows represent activation; red T- bars represent inhibition; double arrows represent multiple steps. Keys: 11-DHC, 11-dehydro-corticosterone; 11β-HSDs, 11β-hydroxysteroid dehydrogenases; CS, corticosterone; Ibil, indirect bilirubin; NA, nicotinate; NAD, nicotinamide adenine dinucleotide; NADP, nicotinamide adenine dinucleotide phosphate; NAM, nicotinamide; NAM *N*-Oxide, nicotinamide *N*-oxide; NMN, nicotinamide mononucleotide; NSS, not statistically significant, Thr, threonine; Trp, tryptophan; Val, valine; XDH, xanthine dehydrogenase; XO, xanthine oxidase.

Metabolomic analyses revealed that acute restraint stress reduced the levels of NAD and NADP in the liver (Figure 5) and increased the levels of nicotinate and nicotinamide *N*-oxide in the urine 24 h post-stress in the male rats (Figure 6). Intracellular NAD/NADH and NADP/NADPH redox pairs play essential roles in various physiological reactions. Under normal conditions, the intracellular levels of NAD(P) remain relatively constant, as they can be recycled between their oxidized and reduced forms [19]. The decline in NAD and NADP levels in the liver indicates a deficiency of NAD(P) in stressed rats (Figure 9). Nicotinate is the precursor for the de novo biosynthesis of NAD [20]. The increase in urinary nicotinate suggests a decrease in the precursor available for NAD synthesis in the body. Nicotinamide *N*-oxide is an oxidative product of nicotinamide [21] and can be reduced back to nicotinamide (a precursor for the NAD salvage pathway) by NAD(P)H-dependent aldehyde oxidase [22,23]. In stressed rats, NAD(P) deficiency may disrupt the reduction process, impairing the conversion of nicotinamide *N*-oxide back to nicotinamide, which is essential for the NAD salvage pathway. The rise in urinary nicotinamide *N*-oxide and nicotinate indicates that the decreased NAD(P) levels following restraint stress may result from the depletion of key precursors necessary for NAD synthesis.

### 4.1. A Possible Link Between NADP Deficiency and Glucose Metabolism

Our results indicate that restraint stress significantly decreased serum glucose levels in the male rats (3.37 ± 0.27 mmol/L) compared to the non-stressed control group (4.93 ± 0.58 mmol/L), as evident from Table 1 (*p* < 0.001). Furthermore, metabolomic analysis revealed a consistent decrease in glucose levels not only in the serum but also in the brain and plasma of stressed rats, as shown in Table 2. Additionally, we observed reduced brain glycogen levels in stressed rats, as detailed in Table 2. This widespread reduction in glucose levels suggests that stress disrupts energy metabolism across multiple organs, potentially impacting overall physiological function. The liver plays a critical role in the regulation of glucose homeostasis. A previous report showed that NADPH-dependent 11β-hydroxysteroid dehydrogenase type 1 (11β-HSD1) is an important hepatic glucose output regulatory factor [24]. One possible explanation for the decreased glucose level in the brain and plasma of stressed rats is that the decreased availability of NADPH affects the reductase activity of 11β-HSD1 and blunts the hepatic glucose output (Figure 9).

Previous research has emphasized the pivotal role of glycogen turnover in the pathophysiology of mental disorders [25]. Given these findings, several intriguing questions arise: Could stress-induced hypoglycemia trigger the mobilization of brain glycogen, ultimately leading to a decrease in brain glycogen content? Furthermore, is this reduction in brain glycogen associated with the observed decrease in the brain-to-body weight ratio in stressed rats, as depicted in Figure 1? These questions warrant further investigation to better understand the complex interplay between stress, energy metabolism, and mental health.

### 4.2. A Possible Link Between NADP Deficiency and Heme Metabolism

One significant change was the rise in Ibil in blood biochemical tests. The serum Ibil level will be elevated when bilirubin, from the destruction of erythrocytes, is beyond the liver’s capacity to convert it into bilirubin diglucuronide. Early studies reported that serum bilirubin levels correlate to the severity of psychiatric disorders, including depression and schizophrenia [26,27]. NADPH is required to maintain the antioxidant protection system of erythrocytes [28], and acute restraint stress induces a sustained increase of oxidative status in erythrocytes [29]. One possible explanation for the rise in Ibil is that decreased NADPH levels in the stressed rats lead to an increase in NADPH-dependent hemolysis (Figure 9). The results of our metabolomic analyses also support this notion (Table 2).

Threonine, valine, and succinate are precursors of succinyl-CoA for heme synthesis. Hemolysis would stimulate a compensatorily increased heme synthesis that might lead to decreased levels of threonine and valine in the blood and succinate in urine (Figure 9).

### 4.3. A Possible Link Between NAD Deficiency and Uric Acid Metabolism

The oxidation of xanthine and hypoxanthine produces uric acid by XOR, which is then converted to allantoin and subsequently to allantoic acid. The result of XDH activity indicated a decrease in XDH activity in the stressed group (Table 3, Supplementary Figures S3 and S4). As a NAD-reducing enzyme, the activity of XDH depends on NAD as a cofactor. A deficiency in NAD would reduce the catalytic activity of XDH [30]. In humans, uric acid is the final product of purine metabolism, whereas in rats, it can be further metabolized into allantoin and allantoic acid. The significant decreases of allantoic acid and allantoin in tissues (Table 2) and the non-statistically significant decrease in serum uric acid (Table 1) suggest that NAD deficiency affected the catalytic efficiency of NAD-dependent XDH in converting xanthine to uric acid (Figure 9).

In addition, our data demonstrate that the clinical correlation between depressive symptoms and serum uric acid levels can be reproduced in the male rat model [5,6], indirectly supporting the notion that restraint stress is a valid method for studying a depressive phenotype. However, it is crucial to acknowledge the inherent limitations of animal experimental models, particularly when considering the physiological and metabolic discrepancies between rats and humans. Notably, significant differences exist in the metabolic pathways of certain substances. For instance, uric acid serves as a terminal metabolic product in humans, whereas in rats, it undergoes further metabolism into allantoin and allantoic acid. This fundamental distinction could potentially elucidate why reductions in uric acid levels were not statistically significant in the male rats subjected to stress, thereby highlighting the intricate nature of such comparisons. Moreover, the specific conditions under which our study was conducted, specifically utilizing an acute restraint stress model, may not adequately capture the multifaceted characteristics of human depression. This limitation underscores the need for caution in extrapolating findings from animal models to human conditions. Consequently, further research endeavors are imperative to validate our results across a broader spectrum of models and under diverse experimental conditions. Such endeavors will not only strengthen the robustness of our findings but also contribute to a more comprehensive understanding of the underlying mechanisms involved in stress-related disorders.

## 5. Conclusions

Significant differences were observed in 30 metabolites (Table 2) between the non-stressed and stressed groups, including regulation of the NAD(P) pool, glucose homeostasis, heme biosynthesis and degradation, and uric acid production and metabolism, among others. Since liver NAD and NADP levels were significantly decreased, while urinary nicotinamide *N*-oxide and nicotinate were significantly increased in the stressed group (Table 2), we conclude that the depletion of NAD(P) precursors occurs in response to restraint stress. Furthermore, the functional analysis of XOR, the rate-limiting enzyme in purine catabolism, showed that restraint stress had limited effects on gene expression and protein abundance, but significantly reduced XOR activity without affecting XO activity. Our findings offer experimental evidence regarding the regulatory effect of NAD on serum uric acid production in an acute restraint-stress-induced male rat model of depression. Further research is needed to explore whether NAD deficiency is a widespread mechanism in stress-induced depression and to assess the potential of NAD replenishment in promoting metabolic recovery from this condition.

## Figures and Tables

**Table 1:** Serum biochemical parameters of non-stressed and stressed rats.

Group	Non-Stressed	Stressed
TP *^a^*	43.65 ± 7.36 *^b^*	43.73 ± 2.22
Alb	25.20 ± 3.43	24.21 ± 0.74
Glo	18.45 ± 4.15	19.51 ± 1.52
Alb/Glo	1.39 ± 0.17	1.25 ± 0.06
Tbil	2.45 ± 1.04	1.97 ± 0.25
Dbil	1.45 ± 1.14	0.69 ± 0.13
Ibil	1.00 ± 0.20	1.28 ± 0.15 *
ALT	39.50 ± 14.60	36.40 ± 9.60
AST	134.50 ± 43.02	145.28 ± 24.55
AST/ALT	3.57 ± 1.06	4.07 ± 0.49
GGT	0.10 ± 0.04	0.18 ± 0.26
ALP	231.33 ± 59.86	252.18 ± 54.72
TG	1.07 ± 0.78	0.93 ± 0.48
TC	1.86 ± 0.34	1.79 ± 0.11
HDL	0.51 ± 0.09	0.53 ± 0.05
LDL	0.15 ± 0.05	0.15 ± 0.02
Glc	4.93 ± 0.58	3.37 ± 0.27 **
LDH	1070.77 ± 410.95	1395.87 ± 183.29
BUN	3.82 ± 1.56	4.48 ± 0.84
Cn	39.33 ± 12.97	44.13 ± 2.80
Bun/Cn	0.10 ± 0.02	0.10 ± 0.01
UA	164.67 ± 78.32	104.58 ± 26.78
TBA	33.60 ± 19.64	52.73 ± 47.84

*^a^* TP, total protein (g/L); Alb, albumin (g/L); Glo, globulin (g/L); Tbil, total bilirubin (μmol/L); Dbil, direct bilirubin (μmol/L); Ibil, indirect bilirubin (μmol/L); ALT, alanine aminotransferase (U/L); AST, aspartate aminotransferase (U/L); GGT, gamma glutamyltransferase (U/L); ALP, alkaline phosphatase (U/L); TG, triglycerides (mmol/L); TC, total cholesterol (mmol/L); HDL, high-density lipoprotein (mmol/L); LDL, low-density lipoprotein (mmol/L); Glc, glucose (mmol/L); LDH, lactate dehydrogenase (U/L); BUN, blood urea nitrogen (mmol/L); Cn, creatinine (μmol/L); UA, uric acid (μmol/L); TBA, total bile acid (μmol/L). *^b^* Each value represents the mean ± S.D. * represents significant differences between the two groups as determined by Student’s *t*-test using SPSS (* *p* < 0.05; ** *p* < 0.001).

**Table 2:** An overview of metabolic alterations in rat body fluids and tissues under stress versus non-stress conditions.

Discriminatory Metabolites	Brain N-S48 *^a^*	Kidney N-S48	Liver N-S48	Lung N-S48	Spleen N-S48	Plasma N-S48	Urine N-S24	Urine N-S48
Acetone	-	-	-	-	-	-	−0.774 *^b^*	-
Adenosine	-	-	-	0.773	-	-	-	-
Allantoic acid	-	-	-	−0.815	-	-	-	-
Allantoin	−0.846	-	−0.758	-	-	-	-	-
Alpha-glucose	−0.795	-	-	-	-	−0.904	-	-
Beta-glucose	-	-	-	-	-	−0.871	-	-
Creatine	0.868	-	-	-	-	-	-	-
Creatinine	0.795	-	-	-	-	-	-	-
Ethanolamine	-	0.762	-	-	-	-	-	-
γ-Aminobutyrate	0.766	-	-	-	-	-	-	-
Glutamate	0.797	-	−0.774	-	-	-	-	-
Glycerophosphocholine	−0.791	-	-	-	-	-	-	-
Glycogen	−0.791	-	-	-	-	-	-	-
Guanosine	-	-	−0.832	0.830	-	-	-	-
Lactate	0.862	-	-	-	-	0.907	-	-
Leucine	−0.803	-	-	-	-	-	-	-
*N*-acetylaspartate	0.901	-	-	-	-	-	-	-
NAD^+^	−0.767	-	−0.834	-	-	-	-	-
NADP^+^	-	-	−0.822	-	-	-	-	-
Nicotinamide *N*-oxide	-	-	-	-	-	-	0.767	-
Nicotinate	-	-	-	-	-	-	0.839	-
Pantothenate	-	0.851	-	-	-	-	-	-
Phosphocholine	−0.796	-	-	-	-	-	-	-
Phosphoethanolamine	−0.882	-	-	-	-	-	-	-
Succinate	-	-	-	-	-	-	-	−0.806
Taurine	−0.797	-	-	-	-	-	-	-
Threonine	−0.771	-	-	-	-	-	-	-
Tyrosine	-	0.791	-	-	-	-	-	-
Uracil	-	0.786	-	-	-	-	-	-
Valine	−0.791	-	-	-	-	-	-	-

*^a^* N-S48, stress-treated group at 48 h post-stress time point compared with the corresponding non-stress control; N-S24, stress-treated group at 24 h post-stress time point compared with the corresponding non-stress control. *^b^* Correlation coefficients (r); positive and negative signs indicate positive and negative correlations in the concentrations, respectively. A correlation coefficient of |r| > 0.755 was used as the cutoff value for statistical significance. “-” means the correlation coefficient |r| is less than the cutoff value.

**Table 3:** Serum xanthine dehydrogenase activities of non-stressed and stressed rats.

	Xanthine Dehydrogenase Activity
Non-stressed group	0.246 U/mL
Stressed group	0.171 U/mL

## Data Availability

The original contributions presented in the study are included in the article/Supplementary Materials; further inquiries can be directed to the corresponding authors.

## Supplementary Materials

The following supporting information can be downloaded at https://www.mdpi.com/article/10.3390/metabo14120660/s1, Figure S1: Photomicrographs of representative sections of the kidney (A,B), lung (C,D), liver (E,F), and spleen (G,H) from non-stressed (A,C,E,G) and stressed (B,D,F,H) rats.; Figure S2: PCA score plots (PC1 vs. PC2) derived from the ^1^H NMR data of tissue samples (extracted from brain, kidney, liver, lung, and spleen) and body fluid samples (plasma and urine); Figure S3: Effect of acute restraint stress on serum xanthine oxidoreductase activity; Figure S4: Effect of acute restraint stress on serum xanthine oxidoreductase activity; Table S1: Body weight (gram) of each rat just before sacrifice with mean ± S.D. and *p*-value; Table S2: The ^1^H NMR data of the metabolites in body fluid samples (plasma and urine) and tissue samples (extracted from brain, kidney, liver, lung, and spleen).
