# Supplementary material for: Disrupted NAD(P) Metabolism and Xanthine Dehydrogenase in a Stress-Induced Rat Model of Depression: NMR Metabolomics Insights
Source: Metabolites. 2024 Nov 27;14(12):660. doi: 10.3390/metabo14120660 (PMC11676094; doi:10.3390/metabo14120660)
Supplement: Supplementary file 1 [file metabolites-14-00660-s001.zip › metabolites-3294323-supplementary.pdf]

Supplementary Material
Disrupted NAD(P) Metabolism and Xanthine Dehydrogenase in a Stress-Induced Rat Model of Depression: NMR Metabolomics Insights

Songjiao Chen [1], Jumeng Wei [1,], Yongchi Wang [2], Yidan Yao [2], Haibo Wang [2], Jie Peng [3,*], Jinquan Li [2,*]*

[1] College of Resources and Environment Science, Anhui Science and Technology University, Fengyang, 233100, Anhui, China.

[2] School of Life and Health Science, Anhui Science and Technology University, Fengyang, 233100, Anhui, China.

[3] Innovative Institute of Animal Healthy Breeding, College of Animal Science and Technology, Zhongkai University of Agriculture and Engineering, Guangzhou, Guangdong, 510225, China.

[*] Correspondence to Dr. Jumeng Wei, Dr. Jie Peng and Dr. Jinquan Li

E-mail: weijm@ahstu.edu.cn (J. Wei)

E-mail: pengjie@zhku.edu.cn (J. Peng)

E-mail: lijinquan@ahstu.edu.cn (J. Li)

School of Life and Health Science, Anhui Science and Technology University, No. 9 Donghua Road, Fengyang, Anhui 233100, China

Tel +86 0550 673 2024

Supplementary Figures

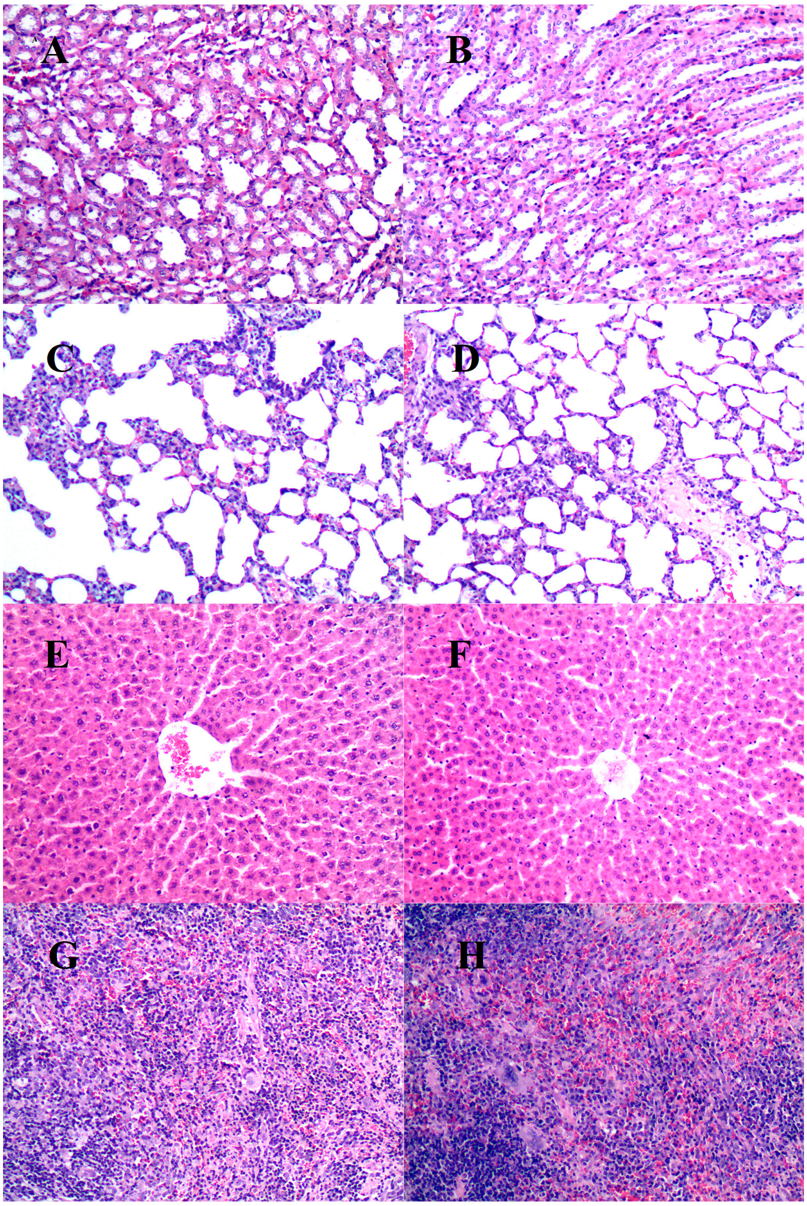

Figure S1. Photomicrographs of representative sections of the kidney (A and B), lung (C and D), liver (E and F) and spleen (G and H) from non-stressed (A, C, E and G) and stressed (B, D, F and H) rats. The tissue sections were stained with hematoxylin-eosin and observed under a 100× microscope. No distinct histological changes were observed in all tissues examined.

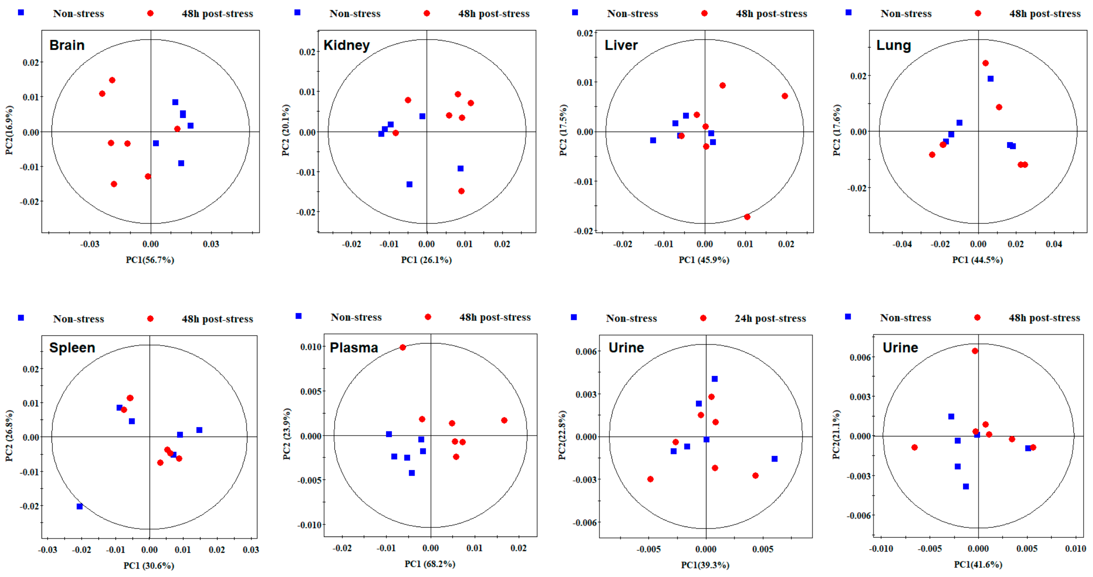

Figure S2. PCA score plots (PC1 vs. PC2) derived from the ^{1}H NMR data of tissue samples (extracted from brain, kidney, liver, lung, and spleen) and body fluid samples (plasma and urine). Non-stress, non-stressed group; 48h post-stress, 48h post-stress group; 24h post-stress, 24h post-stress group.

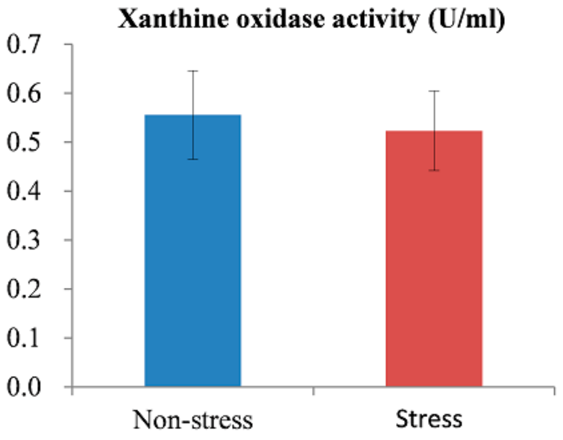

Figure S3. Effect of acute restraint stress on serum xanthine oxidoreductase activity. Non-stress, non-stressed group; Stress, 48h post-stress group.

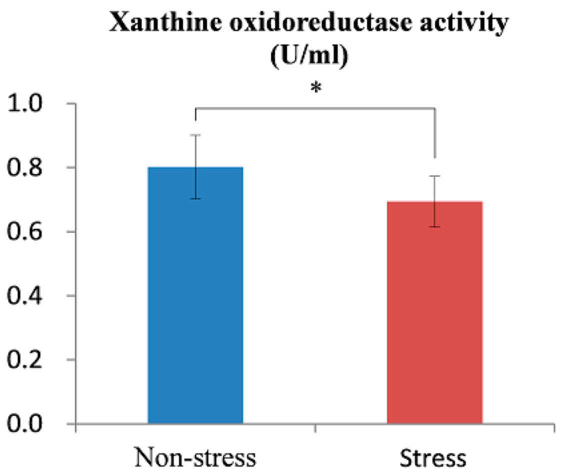

Figure S4. Effect of acute restraint stress on serum xanthine oxidoreductase activity. Non-stress, non-stressed control group; 48h post-stress, 48h post-stress group. * represents significant differences between two groups as judged by Student's t-test using SPSS (*P<0.05).

Supplementary Tables

Table S1 Body weight (gram) of each rat just before sacrifice with mean ± S.D. and *P*-value

Groups	Body weight (gram)							Mean ± S.D. (gram)	*P*-Value
Non-stress	275	276	276	284	271	277	/	276 ± 4	0.136
Stress	274	275	271	274	275	276	270	273 ± 2	

Non-stress, non-stressed group; Stress, 48 h post-stress group.

Table S2 The ^{1}H NMR data of the metabolites in body fluid samples (plasma and urine) and tissue samples (extracted from brain, kidney, liver, lung, and spleen)

Discriminatory metabolites	Abbreviation	^{1}H Shift (multiplicity^{a})	Moiety	Sample
Acetone	Act^{b}	2.23(s)	CH_3	Urine
Adenosine	Ade	4.30(dd); 4.44(dd); 6.07(d); 8.26(s); 8.34(s)	CH(5); CH(4); CH(2); N-CH=N; N-CH'=N	Lung
Allantoate	Al	5.34(m)	CH	Lung
Allantoin	All	5.39(s)	CH	Brain, Liver
Alpha-glucose	α-Glc	3.42(t); 3.54(dd); 3.71(t); 3.74(m); 3.84(m); 5.24(d)	CH(4); CH(2); CH(3); CH(6); CH(5&6′); CH(1)	Brain, Plasma
Beta-glucose	β-Glc	3.25(dd); 3.41(t); 3.46(m); 3.49(t); 3.72(dd); 3.90(dd); 4.65(d)	CH(2); CH(4); CH(5); CH(3); CH(6); CH(6'); CH(1)	Plasma

Creatine	Cr	3.04(s); 3.93(s)	CH3; CH2	Brain
Creatinine	Cn	3.05(s); 4.06(s)	CH3; CH2	Brain
Ethanolamine	EA	3.14(t); 3.83(t)	CH2NH2; CH2OH	Kidney
Gama-aminobutyrate	GABA	1.90(m); 2.30(t); 3.02(t)	α-CH2; β-CH2; γ-CH2	Brain
Glutamate	Glu	2.05(m); 2.13(m); 2.35(m); 3.78(dd)	β-CH; β-CH'; γ-CH2; α-CH	Brain, Liver
Glycerophosphocholine	GPC	3.23(s); 3.68(m); 3.96(m); 4.33(m)	CH3; N-CH2&HO-CH2; CH&O-CH2; P-O-CH2	Brain
Glycogen	Glg	3.63(m); 3.83(m); 3.96(m); 5.40(m)	CH(3, 5, 29); CH(6, 23, 41); CH(4, 9); CH(2, 17, 25)	Brain
Guanosine	Gu	5.90(s); 7.99(s)	CH(2); CH(7)	Liver, Lung
Lactate	Lac	1.33(d); 4.11(q)	CH3; CH	Brain, Plasma
Leucine	Leu	0.96(t); 1.70(m)	CH3; CH2&γ-CH	Brain
N-acetylaspartate	NAA	2.02(s); 2.50(m); 2.70(m); 4.39(m); 7.92(d)	CH3; α-CH; α-CH'; β-CH	Brain
NAD+	NAD	4.49(dd); 4.55(m); 6.04(d); 6.09(d); 8.18(s);8.21(m); 8.43(s); 8.84(d); 9.15(d); 9.34(s)	CH(29); CH(4, 26); CH(2); CH(28); CH(12); CH(38); CH(7); CH(39); CH(37); CH(35)	Brain, Liver
NADP+	NADP	6.05(d); 6.15(d); 8.16(s); 8.42(s); 8.83(d); 9.11(d); 9.29(s)	CH(32); CH(2); CH(12); CH(7); CH(41); CH(43); CH(39)	Liver
Nicotinamide *N*-oxide	NAM *N*-oxide	7.59(dd); 8.24(dd); 8.72(dd); 8.94(s)	CH(5); CH(4); CH(6); CH(2)	Urine
Nicotinate	NA	7.51(m); 8.26(m); 8.61(dd); 8.94(d)	CH(5); CH(4); CH(6); CH(2)	Urine
Pantothenate	Pan	0.88(s); 0.92(s)	CH3(Ⅰ); CH3(Ⅱ)	Kidney
Phosphocholine	PC	3.22(s); 3.59(m); 4.17(m)	CH3; N-CH2; O-CH2	Brain
Phosphoethanolamine	PEA	3.23(t); 3.99(m)	NCH2; OCH2	Brain
Succinate	Suc	2.41(s)	CH	Urine
Taurine	Tau	3.27(t); 3.43(t)	CH2SO3; NCH2	Brain
Threonine	Thr	1.33(d); 3.59(d); 4.25(m)	CH3; α-CH; β-CH	Brain
Tyrosine	Tyr	6.89(d); 7.19(d)	m-CH; o-CH	kidney

Uracil	Ura	5.80(d); 7.53(d)	CH(5); CH(6)	Kidney
Valine	Val	0.99(d); 1.04(d)	γ-CH3; γ-CH´3	Brain

[a] Multiplicity: m, multiplet; br, broad; s, singlet; d, doublet; t, triplet; q, quartet; dd, doublet of doublets. [b] The abbreviation is the same as used in Figure 4, 5 and 6.
